# 3D Exploration and Navigation with Optimal-RRT Planners for Ground Robots in Indoor Incidents

**DOI:** 10.3390/s20010220

**Published:** 2019-12-30

**Authors:** Noé Pérez-Higueras, Alberto Jardón, Ángel Rodríguez, Carlos Balaguer

**Affiliations:** Robotics Lab, Department of Systems Engineering and Automation, University Carlos III of Madrid, Avda. Universidad, 30, Leganés, 28911 Madrid, Spain; ajardon@ing.uc3m.es (A.J.); balaguer@ing.uc3m.es (C.B.)

**Keywords:** mobile robotics, search and rescue robots, path planning, navigation, exploration, point clouds

## Abstract

Navigation and exploration in 3D environments is still a challenging task for autonomous robots that move on the ground. Robots for Search and Rescue missions must deal with unstructured and very complex scenarios. This paper presents a path planning system for navigation and exploration of ground robots in such situations. We use (unordered) point clouds as the main sensory input without building any explicit representation of the environment from them. These 3D points are employed as space samples by an Optimal-RRTplanner (RRT${}^{*}$) to compute safe and efficient paths. The use of an objective function for path construction and the natural exploratory behaviour of the RRT${}^{*}$ planner make it appropriate for the tasks. The approach is evaluated in different simulations showing the viability of autonomous navigation and exploration in complex 3D scenarios.

## 1. Introduction

The development of autonomous robots capable of operating in dangerous situations for humans like inspection, surveillance or search and rescue, is a topic of intense investigation in the field of Robotics. Some examples of robotic systems devised for real rescue applications are a multi-flipper controlled platform for collapsed environments [1], the team of teleoperated robots employed in the earthquake in Italy in 2012 [2] or a review of terrestrial robotic systems for nuclear environments, like Fukushima, in Reference [3]. It is remarkable that most of the real robotic applications are teleoperated, which indicates the complexity of developing effective autonomous behaviour in such environments.

Autonomous navigation is a complex function for performing the referred tasks. Within such cluttered and diverse situations, robots must address several issues like localization, perception and representation of the environment, or planning optimal paths according to given criteria. Moreover, in the case of ground robots, an assessment of the terrain traversability is required [4].

For perception, 3D range sensors are usually employed. In outdoos tasks, like self-driving cars and 3D lidars (moving 2D lasers to cover a set of layers in different angles) are widely used since they can provide a big amount of accurate range data at almost the 360${}^{\circ }$ around the vehicle at long distances. In other tasks that do not require long ranges, like indoors or in confined environments, cameras with depth perception are used. They are smaller and lighter devices than 3D lidars, and provide point clouds in a shorter field of view and ranges between 0.4 m and 10.0 m.

From data of 3D range sensors, different structures are derived for the representation of the environment (map) and different techniques can be employed to describe the terrain for robot navigation. Common approaches are triangular meshes [5], the analysis through basic shapes obtained by the Tensor Voting technique [6,7], or mainly voxel grids like octomaps [8,9,10]. Other methods capture spatial relationships between measurements of the environment naturally. They are called Hilbert maps [11].

However, the generation and/or maintenance of most of these structures can be computationally expensive and lead to a discretization of the space.

On the contrary, we propose working on the point clouds generated from the sensors since we perform a terrain analysis on-demand on robot-sized areas of the environment; similar to the approach of Krusi et al. [12]. Along with this idea, the use of RRT planners [13] seems to be an appropriate option, since we can directly employ the point cloud as a sampling space for the planner and perform the on-demand surface evaluation on it.

In addition to navigation, the autonomous exploration of an unknown area, as in search and rescue missions, is considered in this work. Generally, the exploration is performed by determining regions called "frontiers." The frontiers are regions on the boundary between known open space (no obstacles) and unexplored space. Therefore, by reaching the frontier regions we have more chance to gain information about the environment (map) [14].

Recently, several advances in exploration for aerial vehicles have been made, some examples are found in References [15,16,17]. However, ground robots are still necessary because of their larger autonomy and their higher payload capacity. In Reference [18], Octomaps are used for ground robot navigation applied to search and rescue missions. Another example of a navigation system for that task is Reference [19]. Moreover, other authors also make use of the exploratory benefits of the sampling-based methods, like RRT planners, for detecting frontiers on 2D grids [20,21,22] or 3D grids [23]. Our approach also takes advantage of the exploratory behavior of RRTs. Nevertheless, we do not need to discretize the space in a voxel structure, since we use the point cloud directly.

In Reference [24] a potential field approach for exploration is proposed. Good simulation results in 2D are presented. The approach is closely linked to a PoseSLAM algorithm since the uncertainty in the robot poses are taken into account to build an entropy information field for exploration and loop closure. In contrast, we propose a different 3D approach that detaches the autonomous exploration from the SLAM problem. Our approach is able to work with any SLAM algorithm able to provide an online point-cloud map. Another approach for 2D environments are presented in Reference [25], which is based on Growing Neural Gas (GNG) networks; or Reference [26], which proposes a safe and efficient navigation algorithm for exploration of planar scenarios.

The approach presented has been devised to assist rescuers in mine incident environments—the system can perform an autonomous exploration of the area, or the rescuer can indicate remotely a goal position to reach. Thus, the contribution of this paper is twofold—(1) a safe and effective path planning system for ground robot navigation in 3D environments, and (2) an autonomous exploration approach that makes use of the previous path planning system.

This paper is structured as follows. First, we describe the path planning approach based on point clouds and RRT${}^{*}$ planners in Section 2.1. Section 2.2 presents the exploration approach based on the previous path planner. Then, Section 3 shows the results of a set of experiments in simulated environments. Finally, Section 4 summarizes the paper’s contribution and outlines future work.

## 2. Methods and Algorithms

The methods and algorithms that have been developed are described here. First, the path planning system is presented. Second, the exploration approach based on the previous path planner is explained.

### 2.1. RRT*—Based Path Planning in 3D Environments

#### 2.1.1. RRT* Planning on Point Clouds

RRT planners can cope with continuous state and action spaces and kinodynamic constraints They can be easily extended to spaces with higher dimensions [13]. In particular, we make use of Optimal RRT planners (RRT${}^{*}$) [27]; which, unlike regular RRTs, are asymptotically optimal. They explore the configuration space to obtain optimal paths on cost spaces. Particularly, they sample the configuration space randomly and create a tree towards the goal based on a cost function evaluated for each node candidate of the tree.

In this work, we do not build and maintain any surface map, like polygonal meshes or octomaps to assess the traversability of the terrain. Instead, we make use of (unordered) point clouds obtained from 3D range sensors directly. Thus, we avoid the computational cost of maintaining such structures and the inherent space discretization that can lead to the loss of information.

We follow a similar approach to the work of Krüsi [12], in which nearest-neighbour search over the 3D space points is performed in particular robot-sized areas.

**Figure 1:**
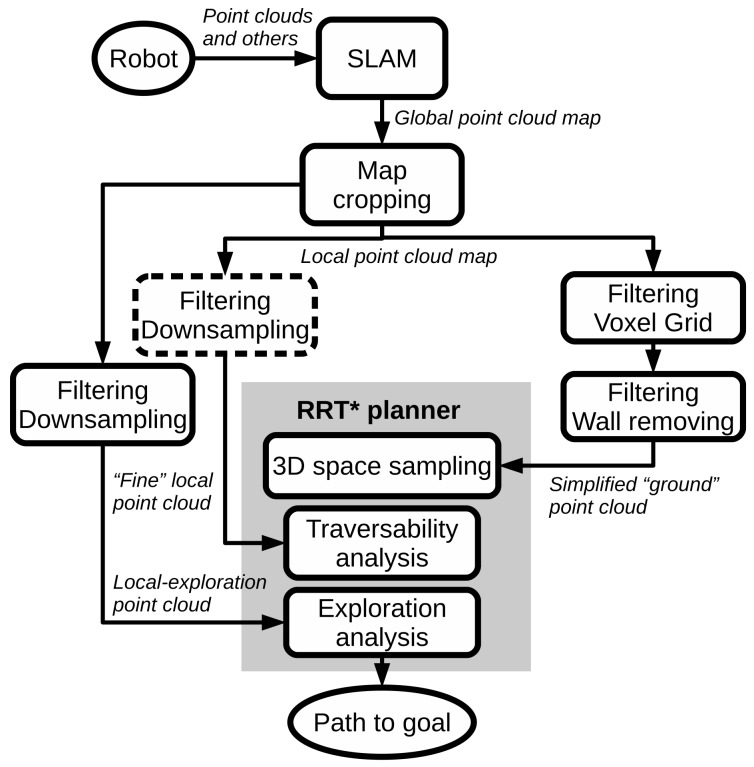
General diagram of the exploration and path planning system.

**Figure 2:**
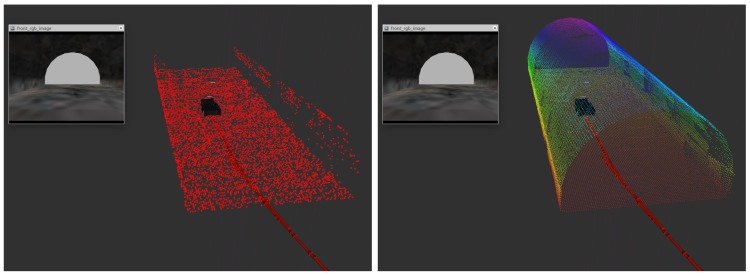
Pointclouds examples. Left: local point cloud used as sampling space for the RRT${}^{*}$ planner in red color. Right: local point cloud employed by the RRT${}^{*}$ for traversability analysis (multicolor).

Figure 1 shows a general diagram of the proposed exploration and path planning system. We propose a scheme in which two different point clouds are provided to the RRT${}^{*}$ planner—one as a sampling space, and a second one for traversability analysis. For exploration analysis, the global point cloud is downsampled and cropped to a local area a bit bigger than the one employed for traversability, as explained in Section 2.2. Moreover, Figure 2 presents a simple example of the two different point clouds in a tunnel environment employed by the path planning system.

Initially, the general point cloud map is cropped to a local robot-centred point cloud of size 10 m × 10 m. The reason behind is that we are employing short-range sensors (5–6 m) in indoor environments so that we do not need to evaluate regions farther than that distance. Furthermore, that sets a boundary of the size of the point cloud to be assessed and, therefore, the computation burden remains always controlled.

#### 2.1.2. 3D Sampling Space for RRT* Planning

As sampling space for the planner, we employ the points of the local point cloud instead of sampling for the whole 3D space. However, unlike [12], we perform a filtering process over this point cloud. Firstly, a voxel grid structure is employed for downsampling the point cloud (in this paper we use voxels of 5 cm${}^{3}$). Then, Principal Component Analysis (PCA) is used for computing the surface normals based on the covariance matrix of the *N* points contained in each voxel $V_{i}$.

With those data, the surface orientation is obtained and, therefore, we can filter out vertical surfaces like walls and the ceiling, by setting boundaries for the pitch and roll angles, δ and β respectively, of the surface:

(1) $$\begin{array}{c} \left|\delta \right|\leq \delta_{max}\in R_{>0} \end{array}$$

(2) $$\begin{array}{c} \left|\beta \right|\leq \beta_{max}\in R_{>0}. \end{array}$$

Thus, we prevent the RRT${}^{*}$ planner from sampling from invalid areas like walls and ceilings, and consequently, to waste computing time in evaluating them.

It is worth noting that these filters and pre-evaluations are executed in parallel to the RRT planning, which takes the newest processed data available, and therefore, it is not a sequential process.

#### 2.1.3. Terrain Analysis

The traversability analysis of the surface employed by the RRT${}^{*}$ planner is based on the local point cloud, according to Figure 1. Also, a visual example can be seen in the right image of Figure 2. Depending on the complexity of the scenario, the local point cloud can be downsampled or not (dotted square of Figure 1). This has a clear effect on the RRT${}^{*}$ computation time as will be discussed in Section 3.

For each sample taken by the planner, the points of the local point cloud that lies inside a sphere around the sample are analyzed. This sphere has the size required to circumscribe the robot shape, so the radius of the sphere, *r*, is a required parameter. It is noteworthy that we use in this work a sphere for the sake of generality of the algorithm but it could be changed to other shapes, like a rectangular box, that can fit better the robot dimensions or its touch surfaces.

Based on this analysis, a cost is assigned to the corresponding sample and a new node is added to the tree. Without loss of generality, we can assume that the $RRT^{*}$ cost function for each point *p* can be expressed as a weighted linear combination of a set of *J* functions $f_{j}\left(p\right)$ and weights $\omega_{j}$ defining the task according to:

(3) $$c\left(p\right)=\sum_{j=1}^{J}\omega_{j}f_{j}\left(p\right)=\omega^{T}f\left(p\right).$$

This function $f\left(p\right)={\left[f_{1}\left(p\right),f_{2}\left(p\right),\cdots ,f_{J}\left(p\right)\right]}^{T}$ is based on *J* measurable characteristics that describe the task to be performed and are called features [28]. The cost of a path is then the sum of the cost for all points in it.

The set of features most-employed for terrain analysis are the inclination (pitch δ and roll β), and also the roughness λ of the surface [12]. The roughness can be approximated by the smallest eigenvalue obtained, along with the pitch and roll, through the technique explained in Section 2.1.2.

We also propose the use of another set of secondary features that may help to evaluate the terrain:

- Number of points in the sphere, η. The more points we have in the area the more accurate and reliable the representation of the surface is. In our case, we consider that a value of one point per centimetre in a plane is good density, although this value can be changed according to the particular constraints. Therefore, an upper boundary of number of points has been calculated as $\eta_{max}=\pi *sphere\_radius^{2}*100$. In which the sphere_radius is presented in meters. This boundary is also used for normalization:
  (4) $$\eta =(1.0-num\_points/\eta_{max}).$$
- Distance between the sample $p_{i}$ and the mean of the set of points $\bar{p}$, named as $d_{m}$. If these two points are not close, some areas of the region could be poorly represented by very few points in several cases:
  (5) $$d_{m}=dist_{p_{i}-\bar{p}}/sphere\_radius.$$
- Standard deviation of the point set, σ. A high deviation could indicate that the points are dispersed along the patched region, and therefore, the probability of having voids without points could be smaller.
  (6) $$\sigma =(1.0-stddev/\sigma_{max}).$$

Furthermore, we add another feature based on the distance from the sample to the goal $d_{g}$ in order to reduce the path length. Finally, we have a feature set of seven normalized feature functions $f\left(p\right)=\left[\delta ,\beta ,\lambda ,\eta ,d_{m},\sigma ,d_{g}\right]$. In addition, the set of weights is also normalized such as $\sum_{j=1}^{J}\omega_{j}=1$.

Therefore, for each RRT${}^{*}$ space sample $p_{i}$, the terrain is evaluated following the next steps:

1. Obtaining the points around $p_{i}$ through nearest-neighbours search from the local point cloud.
2. Calculating the pitch, roll and roughness values.
3. If δ,β and λ exceed the boundaries, the terrain is considered invalid, and the remaining steps are skipped.
4. If the terrain is valid, the remaining features ($\eta ,d_{m},\sigma ,d_{g}$) are calculated.
5. The cost for the sample is computed according to (3), and a new node is added to the tree following the regular RRT${}^{*}$ expansion and rewiring process.

### 2.2. 3D RRT*-Based Exploration

The proposed path planner is also employed for the task of autonomous exploration. Unlike other works [21,23], we take advantage of the RRT${}^{*}$ path planner over the point cloud for finding frontier points instead of using a regular RRT over an occupancy grid or a voxel grid.

The RRT family of planners present a natural exploratory behavior of the space. That means the leaves of the tree are potential frontier points that must be analyzed. Our approach also presents the advantage that the paths to the leaves have been optimized regarding the terrain traversability criteria presented in Section 2.1.3. It is clear that in exploration mode, the feature of the distance to the goal is not used in the cost function for terrain assessment.

Once the planner has finished the expansion over the area, the leaves of the tree are evaluated and the most promising one is chosen as goal frontier. Since a detailed analysis of the terrain is not necessary for this evaluation, a local point cloud with lower resolution than the one employed for traversability analysis is used. That saves computation time. Furthermore, the size of this point cloud is a bit bigger than the traversability one since we need to take into account the points around the tree leaves. Finally, after this evaluation, the system returns the corresponding path to that goal.

**Figure 3:**
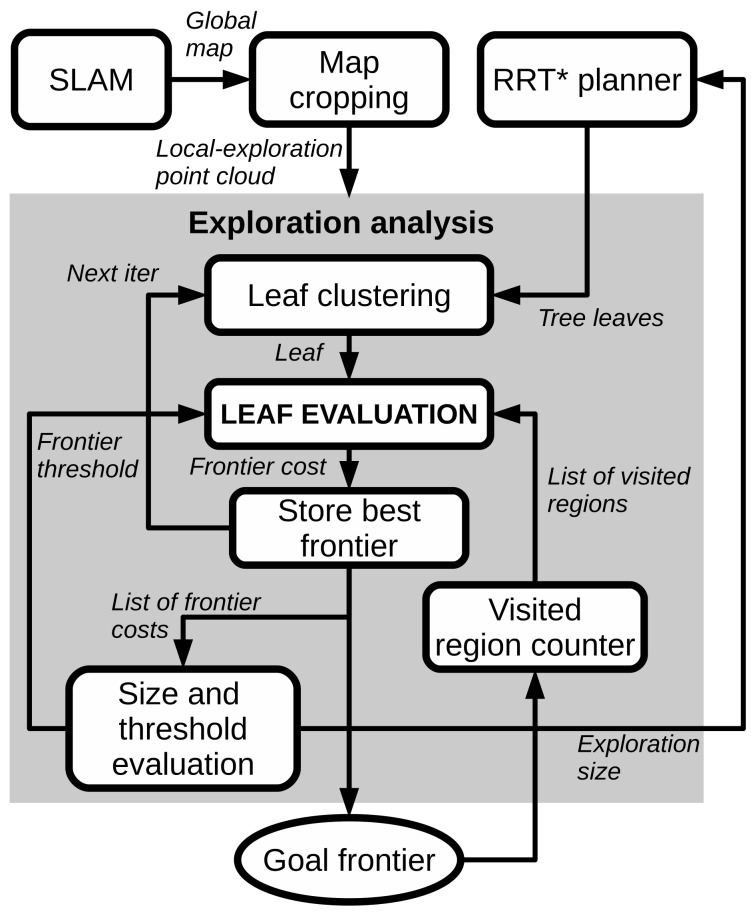
General diagram of the exploration system.

Following the general diagram in Figure 1, a new diagram focused on the Exploration Analysis is presented in Figure 3. The different modules involved in this analysis are explained next.

#### 2.2.1. Leaf Clustering

The number of leaves of the tree can be large and some can be very close to each other in terms of distance. Consequently, we propose performing a clustering of the leaves, as in Reference [21], with the purpose of avoiding unnecessary computations and speed up the process. Nevertheless, instead of taking the mean point of the cluster as a frontier point, a different method is employed. For each cluster detected, the leaf with the minimum path cost is chosen as a potential frontier point and the rest of the leaves are discarded. This way, the robot can reach the area through the most-efficient-path found.

We have used a simple Euclidean clustering, which is defined by three parameters—the radius of search for candidates of clustering, and the minimum and the maximum number of points that can be contained in a cluster. In this work, we have employed a radius of 0.3 m, with a minimum of 2 points and a maximum of 4 points per cluster. With these values, our aim is not to achieve a big reduction in the number of potential frontier points but to simply join those that are very close.

#### 2.2.2. Evaluation of Potential Frontiers

**Figure 4:**
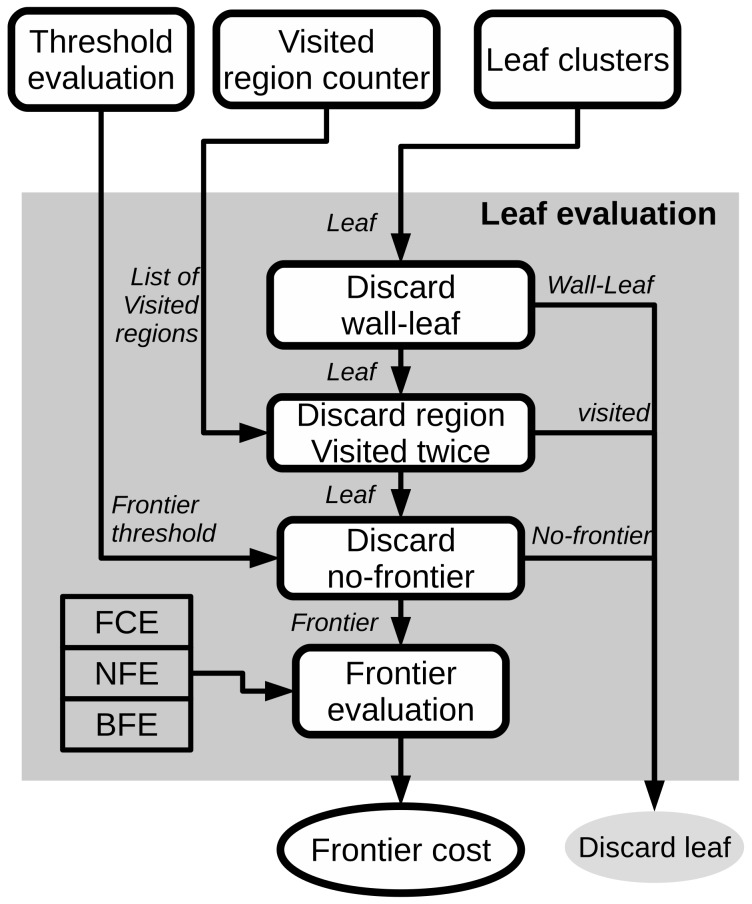
Diagram of the evaluation of potential frontiers.

**Figure 5:**
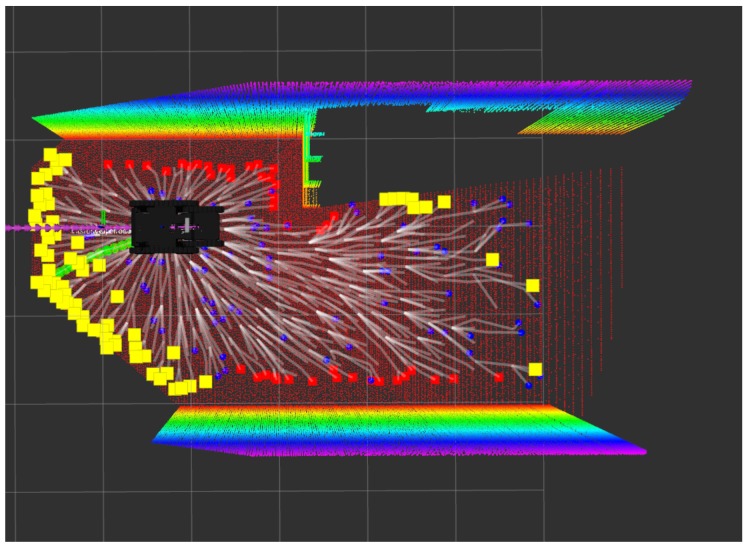
Example of wall-leaf rejection and frontier rejection. The tree leaves in red colour have been discarded as possible frontiers by the wall-leaf detection module. The leaves in yellow color have been accepted as frontiers by the no-frontier rejection module.

Following the diagram presented in Figure 3, after the leaf clustering, a process of evaluation for each selected leaf is performed. Figure 4 shows the different steps and validation checks carried out in that process which are listed next:

1. Wall-leaf rejection.
  Firstly, the position of the leaf in the space is studied. The points inside a sphere of a radius equal to the inflated circumscribed sphere of the robot shape are assessed. The inflation radius has been chosen to be 0.3m. The points are associated with a grid, and the normal vector to the surface formed by the points of each cell is computed. This way, we can count the number of cells of a vertical surface. If this number exceeds a pre-defined threshold, the leaf is considered that is very close to a wall and it is discarded as a possible frontier. An example is shown in Figure 5.
2. Visited region rejection.
  Secondly, the position of the leaf is compared with the position of all the previous visited frontiers. If it is very close to a region that has been visited twice, the leaf is also discarded. To do that, the list of visited regions (with the number of visits) is provided by the counter of visited regions, which is explained in Section 2.2.3. This module plays an important role when there are frontier areas in which it is not possible to gain more information. In those cases, it is able to lead the robot to abandon the areas already explored.
3. No-frontier rejection.
  The third step checks whether the leaf can be considered as a frontier or not. To determine that, the points in a sphere of radius 1.5 m around the leaf (this value can be configured) are counted. Moreover, the standard deviation of the points is also computed. Therefore, the lesser number of points are detected in the area where the more promising the frontier point for exploration is. The number of points (points) and the standard deviation (stddev) are normalized by choosing upper bounds (max_points, max_stddev) employed for the normalization. Finally, a normalized frontier cost $F_{cost}$ for the leaf *p* is computed as:
  (7) $$F_{cost}\left(p\right)=0.7*\frac{points(p)}{max\_points}+0.3*\frac{stddev(p)}{max\_stddev}.$$
  A threshold value $\Delta_{f}$ is set, so that if the frontier cost $F_{cost}$ is higher than the threshold, then the leaf is not considered as a frontier. An example is shown in Figure 5 with a threshold value of $\Delta_{f}=0.4$. The threshold evaluation determines the value of this threshold dynamically as explained in Section 2.2.4.
4. Frontier evaluators.
  Once we have determined the set of tree leaves that are potential frontiers, the selection of the most promising frontier is performed by the frontier evaluators.
  We propose a novel evaluator based on a cost function, $C_{exp}$, that we have called **Cost Function Exploration (CFE)**. This cost is obtained as the weighted sum of the cost of the path to the frontier $C(\zeta_{p_{f}})$, the cost of the frontier $F_{cost}\left(p_{f}\right)$, and a "return" cost $R(p_{f})$, which penalizes the frontiers that are close to areas that the robot has already visited:
  (8) $$C_{exp}\left(p_{f}\right)=\omega_{c}\;C\left(\zeta_{p_{f}}\right)+\omega_{F}\;F_{cost}\left(p_{f}\right)+\omega_{R}\;R\left(p_{f}\right).$$
  Unlike other works based on regular RRTs [21,23], our navigation cost $C(\zeta_{p_{f}})$ includes the terrain evaluation and not only the length of the path to the frontier.
  Finally, the aim of the return cost *R* is to prevent the robot from re-visiting areas where the robot already was close by. This is particularly useful in our setup, which involves tunnel scenarios and the evaluation on a local point cloud instead of the global map. In these cases, the robot is biased to explore forward the tunnel instead of going back.
  The return cost is calculated by computing the distance between the frontier point and the closest point of the trajectory followed by the robot so far. This cost decreases linearly from 1 to 0 in a pre-defined distance range from 0 up to 2 meters:
  (9) $$R\left(p_{f}\right)=max\_dist-d\left(p_{f},t_{r}\right)/max\_dist,$$
  where $d(p_{f},t_{r})$ is the Euclidean distance between the frontier point $p_{f}$ and the closest point of the robot trajectory $t_{r}$. If this distance is greater than the maximum distance threshold (max_dist=2m), the cost returned is zero.
  We also compare this evaluator with two methods widely-used in the literature, as in Reference [22], which have been adapted to work in 3D over point clouds:

- **Nearest Frontiers Exploration (NFE)**. This is an adaptation of the well-known Nearest Frontier approach [14] to 3D point clouds. It is based on proximity criteria by selecting the frontier with the smallest Euclidean distance to the robot ignoring the existence of obstacles.
- **Biggest frontier Exploration (BFE)**. It is based on size criteria. The frontier with less information ($F_{cost}$) is selected as the goal.

#### 2.2.3. Counter of Visited Regions

The counter of visited regions is in charge of storing all the exploration goals selected so far. Moreover, it counts the number of visits that each goal region has received. A visit is counted when the distance between the new frontier goal and any of the previous goals is lower than a distance threshold, $d_{reg}$. In this work, we have employed a distance of 1.5 m. Then, in the leaf evaluation, if the leaf is close to a region visited twice, the leaf is discarded as a possible frontier.

#### 2.2.4. Evaluation of exploration size and frontier threshold

This module analyzes the current exploration situation with the aim of dynamically adapting, firstly, the size of the exploration area. The objective is to save computation time by trying to keep a "small" area for planning and secondly, to change the value of the frontier threshold, which determines the leaves that are considered frontiers according to the frontier costs computed at each iteration of the exploration.

To do that, the module uses a list of the frontier costs computed in the last exploration (cost_array). These costs are compared with a cost boundary κ. The value of the cost boundary is computed regarding the current frontier threshold value ($\kappa =\Delta_{f}-0.05$). Furthermore, the minimum size and the maximum size of the planning area for the RRT${}^{*}$ planner is provided.

The process is described in more detail in Algorithm 1. If the percentage of frontier costs that are equals or higher than the cost boundary is above a pre-defined limit ϵ, the current size of exploration area employed by the RRT${}^{*}$ planner is increased as well as the time employed for planning (from Line 2 to Line 8). If that is not fulfilled, and the current planning size is higher than the minimum size, then the size and the time are decreased (Line 9). Contrary, if the array of costs is empty, which means that we did not find any frontier, the planning size and time is also increased only if the maximum planning size was not reached (Line 12). Finally, if the maximum planning size was reached, the frontier threshold $\Delta_{f}$ is increased with the aim of detecting “smaller” frontiers (Line 14).
**Algorithm 1:** Size-and-threshold evaluator algorithm.
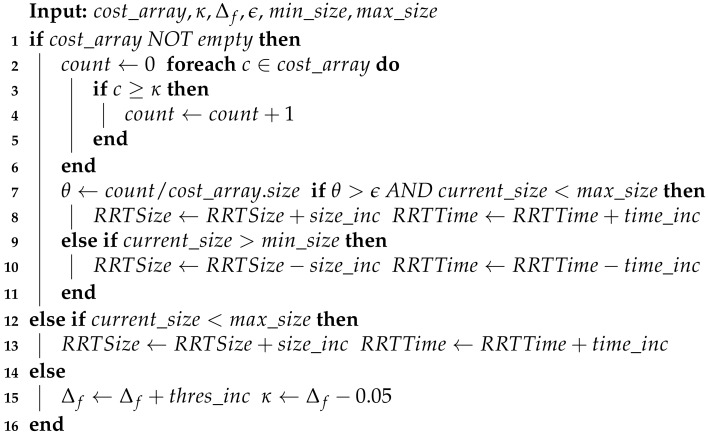


## 3. Results

### 3.1. Implementation and Simulations

The proposed path planning and exploration system has been implemented in C++ under ROS (http://www.ros.org/) [29]. Moreover, the Point Cloud Library (PCL) (http://pointclouds.org/) has been employed for point cloud processing. The developed software is publicly available on Github under BSD license (https://github.com/noeperez/indires_navigation).

It is not part of this work the evaluation or development of SLAM algorithms. Therefore, a publicly available SLAM algorithm that provides an online map based on point clouds has been employed in this evaluation. Specifically, an ICP-based SLAM system which provides a real-time tracker and mapper in 2D and 3D has been used [30,31].

**Figure 6:**
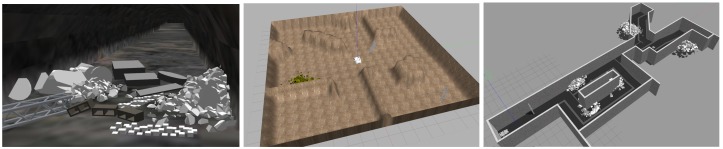
Scenarios for evaluation. Left: tunnel environment with obstacles for navigation (navigation map). Center and right: environments for evaluation of the exploration and navigation (exploration maps 1 and 2 respectively).

For evaluation of the approach, three different environments have been designed and simulated in Gazebo Robot Simulator (http://gazebosim.org/) along with the tracked robot employed in this work. Figure 6 shows a capture of these scenarios. The first environment (left image) is a tunnel with different obstacles in it. The tunnel has a width of around 4 m. The second environment (center image) is a squared area of 17 m × 17 m that presents a bumpy terrain with different ramps and obstacles. The third one (right image), is a set of connected corridors with ramps and fallen rocks inside. The dimensions of the bounding box that limits the space is around 35 m × 14.5 m.

In the real robot we used only an RGB-D camera placed on top of the robot so that we used the same sensor and configuration to perform the evaluation in these experiments. The employed field of view was 80${}^{\circ }$ H × 49.5${}^{\circ }$ V with a distance range of 0.4 m–5.0 m. By using a wider field of view, like 3D lidars, that can cover the 360${}^{\circ }$ around the robot approximately, probably better results can be obtained. However, in indoor and confined environments, like a collapsed tunnel, a large distance range is not required and a lot of unnecessary data would delay the computation of the algorithms. We prove that the performance of the system is still very good under these field-of-view constraints.

All the experiments were executed in a computer with an i7-6700HQ processor and 16GB of RAM.

### 3.2. Navigation Results

The tunnel with obstacles presented in the left image of Figure 6 was employed here to evaluate some aspects of the navigation system as the size and resolution of the point clouds, and the cost function employed for path building.

The robot was placed at the beginning of the tunnel and had to try to reach a goal 5 m away by crossing the obstacles in between. In this scenario, the RRT${}^{*}$ planner is allowed to plan a path for 5 s.

#### 3.2.1. Size of the Point Cloud for Sampling Space

In the work of Krusi [12], the whole point cloud is used as a sampling space for a RRT planner. On the contrary, we use a filtered local point cloud with a fixed size and without walls and ceiling what presents a remarkable impact on the performance of the algorithm.

From 10 path-planning repetitions in the tunnel scenario, we are able to reduce the number of points of the original point cloud in a 93% on average. Besides filtering most of the points of the walls and the ceiling, the point cloud is downsampled to a resolution of 0.05 cm. Thus, we prevent the algorithm from wasting computation time on sampling and evaluating most of the invalid areas. This poses a clear advantage over other approaches, like that in Reference [12], that use the whole point cloud.

Note that the number of points available for sampling must be rich enough to describe the area so that space can be appropriately explored by the tree expansion. Therefore, the resolution of the downsampled space must be properly chosen and very low resolutions must be avoided.

#### 3.2.2. Analysis of the Resolution of the Point Cloud for Traversability Analysis

The resolution of the point cloud employed for terrain analysis also has a significant impact on the algorithm performance. The denser the point cloud is the more accurate the analysis of the surface is. However, the computing burden is also higher since there are more points to be processed.

**Figure 7:**
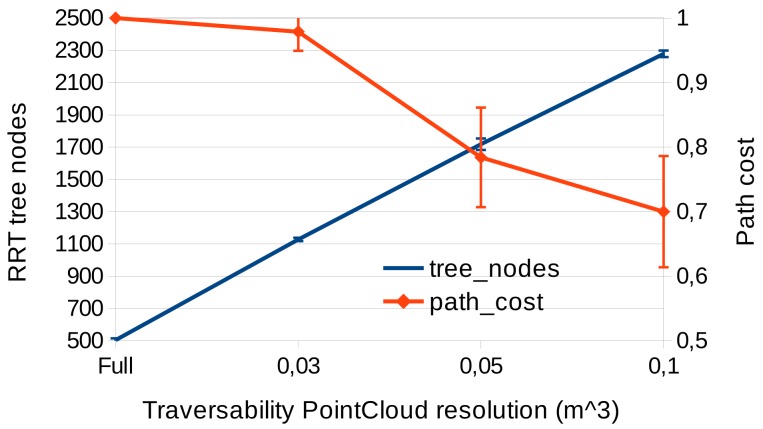
Comparison of pointcloud resolution for traversability versus size of RRT${}^{*}$ tree and path costs in 5secs of planning in the tunnel scenario.

We have performed a comparison of the size of the RRT${}^{*}$ tree (number of tree nodes) and the costs of the paths according to different resolutions of point cloud for traversability. The planning has been repeated 10 times in the tunnel scenario (Figure 7).

On the one hand, with the highest-density point cloud, the algorithm is not able to expand a tree that reaches the goal in the given time (5 s). That is represented with the highest cost possible (1). For a point cloud of 0.03 m${}^{3}$, the algorithm is unable to find a path in some of the 10 cases. In case of using a resolution of 0.1 m${}^{3}$, the costs of the paths are small. Though, the paths obtained overcome almost all the obstacles since they are not properly represented with such low resolution. According to these experiments in the tunnel environment, a resolution of 0.05 m${}^{3}$ seems to be appropriate, although higher resolutions can improve the results if more processing power or time can be given to the planner.

On the other hand, as expected, the lesser number of points to be processed (low resolution) the more speedy the algorithm is (bigger RRT tree). However, the representation of the environment is less accurate if we use fewer points to describe it.

#### 3.2.3. Evaluation of the Cost Function for Path Building

To evaluate the path planning capabilities, we compare a RRT${}^{*}$ cost function based on the regular features for terrain analysis (inclination and roughness) employed in some approaches [5,12], with the proposed augmented cost function described in Section 2.1.3. The weights of the two cost functions have been hand-tuned to the best behavior found. Specifically, we have employed in this work the following weight sets:

- Basic cost function: $\omega_{\delta }=0.4$, $\omega_{\beta }=0.4$, $\omega_{\lambda }=0.2$.
- Augmented cost function: $\omega_{\delta }=0.1$, $\omega_{\beta }=0.1$, $\omega_{\lambda }=0.1$, $\omega_{\eta }=0.175$, $\omega_{d_{m}}=0.175$, $\omega_{\sigma }=0.175$, $\omega_{d_{g}}=0.175$.

We also evaluate the tree expansion according to a different set of threshold values for the pitch, roll and roughness that determine whether a sample is considered valid or not. Three sets for evaluation are considered without regard to the robot characteristics, as presented in Table 1.

**Figure 8:**
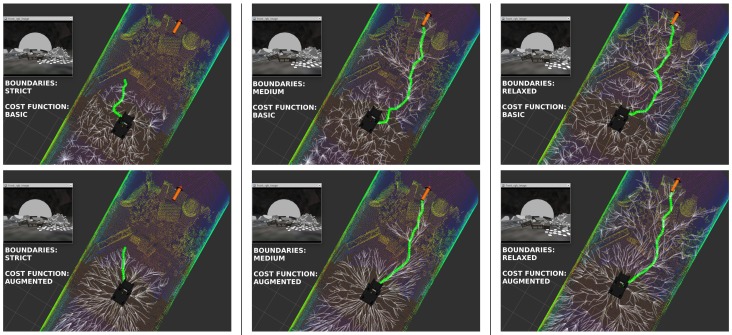
Visual comparison of different tree-expansion boundaries and cost functions.

Figure 8 shows a visual comparison of the paths obtained in the tunnel scenario with different combinations of the boundaries and the cost functions. As can be observed, the proposed cost function improves the paths of the basic cost function. Regarding the tree expansion, the strict boundaries only allow the expansion on the flat floor while the relaxed boundaries allow overcoming almost all the obstacles. Therefore, proper values for the boundaries must be chosen according to the robot capabilities of overcoming obstacles and climbing slopes.

### 3.3. Exploration Results

The exploration process employed in this evaluation is the following. While planning and evaluating, the robot is still on the spot waiting for obtaining a new exploration goal. Therefore, the processing of the next exploration goal is not launched until the robot has reached its previous goal, and consequently, the robot waits there until the new goal has been computed.

The maximum linear velocity that the vehicle can reach is 0.3 m/s, and the maximum angular velocity is 0.6 rad/s. The values of the exploration parameters that have been used can be consulted in Table 2. These parameters have been hand-tuned to the best performance achieved.

#### 3.3.1. Frontier Evaluators Comparison

For comparison we have executed the proposed approach (CFE) and the state-of-the-art methods (NFE and BFE), 3 times for each of the two exploration environments (exploration maps 1 and 2 of Figure 6). We compare the percentage of the surface area that has been explored by each algorithm regarding a ground-truth map that has been built by teleoperating the robot carefully. The duration of the manual exploration has lasted around 20 min for map1, and 25 min for map 2 approximately.

**Figure 9:**
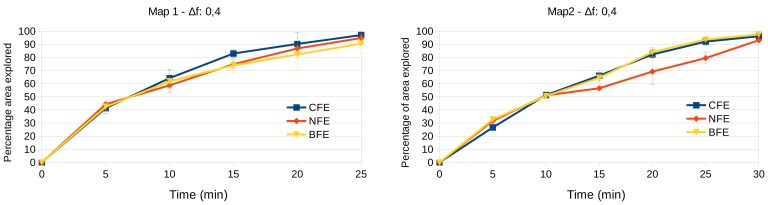
Percentage of ground truth area covered by the proposed approaches. Left: map 1. Right: map 2.

The results are presented in Figure 9 for map 1 and map 2 respectively. The percentage of area explored and their standard deviations along the time elapsed are shown. As can be seen, in both environments, the system is able to lead the vehicle to overcome all the obstacles and cover more than the 90% of the area in a reasonable time in all the cases. Furthermore, the three frontier evaluators reach similar performance in terms of area covered.

However, we can observe some differences regarding the distance traveled by the robot, as stated in Table 3. As expected, the BFE method leads the robot to travel more distance since it always tries to reach the “bigger” frontier detected no matter the distance. On the contrary, the NFE tries to reach the closest frontier at any moment, so, the traveled distance is a bit shorter. Similar distance is reached by the CFE approach, which tries to present a balanced behavior between the other two approaches, according to the chosen weights of its cost function.

The parameters chosen have an important impact on the performance of the approaches. The values of the frontier threshold $\Delta_{f}$, or the minimum and maximum planning sizes are also very relevant as we analyze next.

#### 3.3.2. Frontier Threshold Evaluation

**Figure 10:**
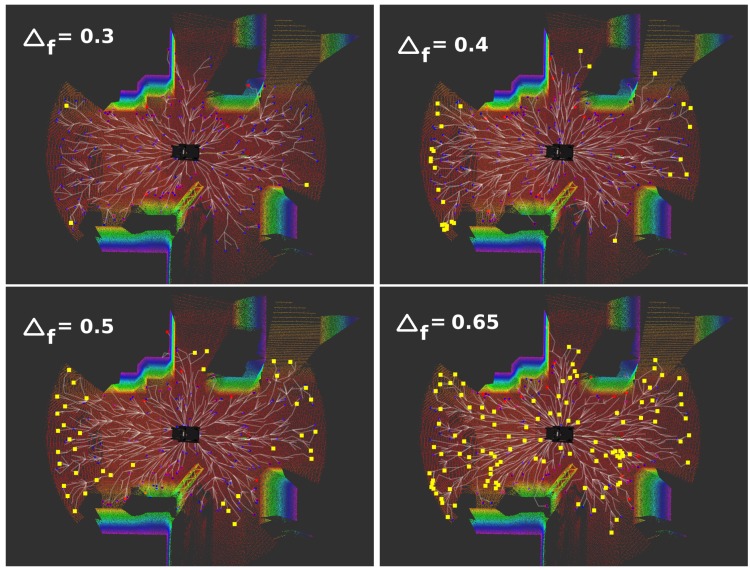
Frontiers detected (yellow squares) according to different values of frontier threshold $\Delta_{f}$.

One of the most important parameters is the frontier threshold $\Delta_{f}$. Figure 10 shows an example of the frontiers detected regarding four different values for $\Delta_{f}$.

As can be observed, it determines how exhaustive the exploration is. If it is very low, only the “biggest” frontiers are detected, and some areas can remain poorly explored. Moreover, if the planning size does not cover the whole area to be explored (most of the cases), the robot can fall into a blocked situation since new frontiers can not be detected. On the contrary, if its value is high, a lot of regions are considered as frontiers and the exploration system will perform a thorough exploration. The dynamical change of this value is also linked to the current planning size, which is also adapted according to the situation. For these reasons, the module of adaptive planning size and frontier threshold is required.

**Figure 11:**
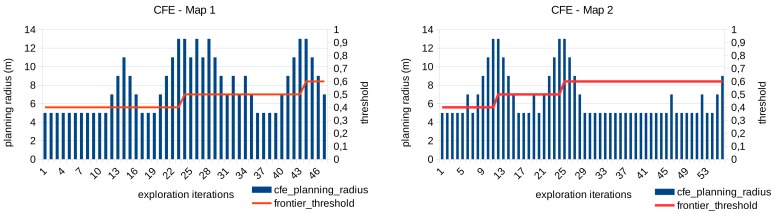
Adaptation of the planning radius and frontier threshold during the exploration of the maps 1 and 2 using the CFE approach.

An example of the functioning of this module is presented in Figure 11. The planning radius around the robot and the frontier threshold are shown for each exploration iteration performed in the exploration maps 1 and 2 respectively. For the shake of clarity, we show only the results of the CFE approach with an initial frontier threshold of $\Delta_{f}=0.4$ since the performance of the module does not depend on the frontier evaluator employed. As can be seen, the planning radius is adapted dynamically with the aim of finding frontier points. When the maximum radius is reached (13 m) and no frontiers are found, the frontier threshold is increased too.

Table 4 shows the results in the percentage of area explored and distance travelled in 25 min of exploration for the three proposed frontier evaluators according to two initial values of $\Delta_{f}$ in the exploration map 1.

On the one hand, in case of using an initial threshold of 0.4, the adaptive-frontier-threshold module increased the threshold during the exploration to reach a final value 0.6 for all the approaches. Moreover, the three proposed approaches present good and very similar metrics.

On the other hand, if the initial threshold is 0.6, many frontiers are detected during the whole exploration and no need of increasing it is required. In this case, the NFE approach worsens its performance. In the same time period, it is able to cover only the 75% of the area since it has a lot of close frontiers to visit. On the contrary, with more frontiers detected, the BFE seems to be affected only in the travelled distance, which has been increased. Finally, the CFE presents a similar performance in both cases. It always tries to choose a “good” frontier according to the cost function from the set of frontiers detected.

Overall, it seems that starting the exploration with a low threshold value and letting the threshold evaluator module to increase when necessary is an appropriate technique. However, this must be fixed according to the complexity of the environment and how exhaustive the exploration is required. Regarding the frontier evaluators, the CFE approach seems to present a slightly better performance than the other two, since it is able to cover the complete area without travelling big distances.

#### 3.3.3. Visited Regions Evaluation

The module of rejection of visited regions can lead the robot to abandon the areas already explored twice and, this way, to avoid block situations in which the robot tries to repeat the exploration of regions where it is not possible to gain more information. This can occur more frequently when the frontier threshold $\Delta_{f}$ is high and a lot regions without a high level of information are considered as frontiers. Hence, instead of staying in the same area trying to gain more information, the robot is pushed to move to other regions.

**Figure 12:**
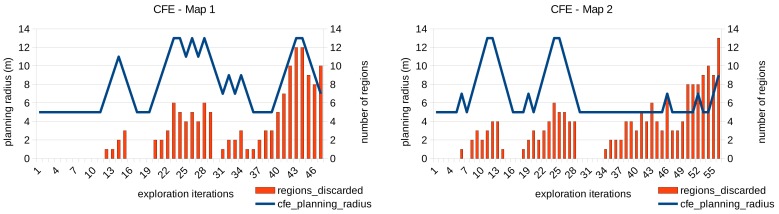
Number of regions discarded for each exploration iteration of the maps 1 and 2 using the CFE approach.

For each exploration iteration, Figure 12 shows the number of regions that were already visited twice and therefore, the close leaves were discarded as possible frontiers. Each region is an sphere with radius $d_{reg}$ = 1.5 m. Again, only the FCE approach is shown since the performance of the visited-region rejection does not depend on the frontier evaluator employed.

As expected, the number of regions discarded is higher when the planning radius is bigger, and also when the area of the given map is almost completely explored.

## 4. Conclusions

This paper presented a navigation and exploration system for ground robots in complex indoor 3D environments like a mine. The approach has a twofold function since it is employed for autonomous path planning and also for exploration.

Some improvements and novelties are included in the path planning regarding the current approaches of the state of the art. The use of different point clouds with bounded sizes and resolutions directly in the different stages of the RRT${}^{*}$ planning present advantages in efficiency computation time.

To employ the expanded RRT${}^{*}$ tree for exploration, which includes feasible paths to the leaves in the 3D environment, seems to be an efficient approach. Unlike regular methods, the proposed approach can dynamically adapt the size of the area employed for planning, which saves computation resources. Moreover, the modules for frontier threshold evaluation and the counter of visited regions can adapt the system to the situations found and to lead the robot to perform a complete exploration in complex 3D environments. Furthermore, the proposed frontier evaluator presents good results as well as the adaptation of the state-of-the-art approaches.

The experiments in simulation in diverse 3D environments present good performance of the different modules and deliver promising results for the application of the system in real robots.

Future work will consider experimentation with real robots and environments and the study of learning approaches that can help to fit the set of different parameters of the modules and the weights of the cost functions.

## Figures and Tables

**Table 1:** Set of boundaries for RRT${}^{*}$ expansion.

	Strict	Medium	Relaxed
**pitch**	0.7	0.87	1.3
**roll**	0.7	0.87	1.3
**roughness**	0.2	0.8	3.0

**Table 2:** Parameters employed in the exploration evaluation.

$\omega_{c}=0.305\;\left[0,1\right]$	$\omega_{F}=0.39\;\left[0,1\right]$	$\omega_{R}=0.305\;\left[0,1\right]$
min_size=5.0m	max_size=13.0m	size_inc=2.0m
$\Delta_{f}=0.4\;\left[0,1\right]$	thresh_inc=0.1[0,1]	time_inc=0.8seg
κ=0.35[0,1]	ϵ=0.85[0,1]	$d_{reg}=1.5\;m.$

**Table 3:** Comparison of distance traveled in the two environments.

Dist. (*m*)	CFE	NFE	BFE
**Map 1**	74.08±5.84	74.59±11.80	79.10±12.84
**Map 2**	87.63±3.01	86.30±9.95	99.98±5.01

**Table 4:** Comparison of exploration in map 1 with different frontier threshold values.

		CFE	NFE	BFE
$\Delta_{f}=0.4$	Exp.(%)	97.00±2.51	94.76±4.47	90.41±7.14
	Dist.(m)	74.08±5.84	74.59±11.80	79.10±12.84
$\Delta_{f}=0.6$	Exp.(%)	90.01±6.47	75.36±3.55	92.63±1.23
	Dist.(m)	72.07±2.89	41.09±2.33	95.42±7.90.

## References

[1] Menna M., Gianni M., Ferri F., Pirri F. Real-time autonomous 3D navigation for tracked vehicles in rescue environments. Proceedings of the 2014 IEEE/RSJ International Conference on Intelligent Robots and Systems.

[2] Kruijff G.J.M., Tretyakov V., Linder T., Pirri F., Gianni M., Papadakis P., Pizzoli M., Sinha A., Emanuele P., Corrao S. Rescue Robots at Earthquake-Hit Mirandola, Italy: A Field Report. Proceeding of the IEEE International Symposium on Safety, Security and Rescue Robotics (SSRR).

[3] Tsitsimpelis I., Taylor C.J., Lennox B., Joyce M.J. (2019). A review of ground-based robotic systems for the characterization of nuclear environments. Prog. Nucl. Energy.

[4] Papadakis P. (2013). Terrain traversability analysis methods for unmanned ground vehicles: A survey. Eng. Appl. Artif. Intell..

[5] Garrido S., Malfaz M., Blanco D. (2013). Application of the fast marching method for outdoor motion planning in robotics. Rob. Auton. Syst..

[6] Liu M., Siegwart R. Navigation on point-cloud-A Riemannian metric approach. Proceedings of the 2014 IEEE International Conference on Robotics and Automation (ICRA).

[7] Liu M. (2016). Robotic online path planning on point cloud. IEEE Trans. Cybern..

[8] Hornung A., Wurm K.M., Bennewitz M., Stachniss C., Burgard W. (2013). OctoMap: An efficient probabilistic 3D mapping framework based on octrees. Auton. Robot..

[9] Song S., Jo S. Online inspection path planning for autonomous 3D modeling using a micro-aerial vehicle. Proceedings of the 2017 IEEE International Conference on Robotics and Automation (ICRA).

[10] Oleynikova H., Taylor Z., Fehr M., Nieto J., Siegwart R. (2016). Voxblox: Building 3D Signed Distance Fields for Planning. arXiv.

[11] Ramos F., Ott L. (2016). Hilbert maps: Scalable continuous occupancy mapping with stochastic gradient descent. Int. J. Rob. Res..

[12] Philipp Krüsi P., Furgale P., Bosse M., Siegwart R. (2016). Driving on Point Clouds: Motion Planning, Trajectory Optimization, and Terrain Assessment in Generic Nonplanar Environments. J. Field Rob..

[13] Lavalle S.M. (1998). Rapidly-Exploring Random Trees: A New Tool for Path Planning. CiteSeerX.

[14] Yamauchi B. A Frontier-Based Approach for Autonomous Exploration. Proceedings of the IEEE International Symposium on Computational Intelligence, Robotics and Automation.

[15] Witting C., Fehr M., Bähnemann R., Oleynikova H., Siegwart R. (2018). History-Aware Autonomous Exploration in Confined Environments Using MAVs. Proceedings of the 2018 IEEE/RSJ International Conference on Intelligent Robots and Systems, IROS 2018.

[16] Song S., Jo S. Surface-Based Exploration for Autonomous 3D Modeling. Proceedings of the 2018 IEEE International Conference on Robotics and Automation (ICRA).

[17] Dang T., Papachristos C., Alexis K. Visual Saliency-Aware Receding Horizon Autonomous Exploration with Application to Aerial Robotics. Proceedings of the 2018 IEEE International Conference on Robotics and Automation (ICRA).

[18] Dornhege C., Kleiner A. (2013). A Frontier-Void-Based Approach for Autonomous Exploration in 3D. Adv. Rob..

[19] Colas F., Mahesh S., Liu M., Siegwart R. 3D Path Planning and Execution for Search and Rescue Ground Robots. Proceedings of the IEEE/RSJ International Conference on Intelligent Robots and Systems (IROS).

[20] Kantaros Y., Schlotfeldt B., Atanasov N., Pappas G.J. Asymptotically Optimal Planning for Non-Myopic Multi-Robot Information Gathering. Proceedings of Robotics: Science and Systems (RSS).

[21] Umari H., Mukhopadhyay S. Autonomous robotic exploration based on multiple rapidly-exploring randomized trees. Proceedings of the 2017 IEEE/RSJ International Conference on Intelligent Robots and Systems (IROS).

[22] Pimentel J.M., Alvim M.S., Campos M.F.M., Macharet D.G. (2018). Information-Driven Rapidly-Exploring Random Tree for Efficient Environment Exploration. J. Intell. Rob. Syst..

[23] Bircher A., Kamel M., Alexis K., Oleynikova H., Siegwart R. (2018). Receding horizon path planning for 3D exploration and surface inspection. Auton. Robot..

[24] Vallvé J., Andrade-Cetto J. (2015). Potential Information Fields for Mobile Robot Exploration. Robot. Auton. Syst..

[25] Wang D., Duan Y., Wang J. (2015). Environment exploration and map building of mobile robot in unknown environment. Int. J. Simul. Proc. Model..

[26] Savkin A., Li H. (2017). A safe area search and map building algorithm for a wheeled mobile robot in complex unknown cluttered environments. Robotica.

[27] Karaman S., Frazzoli E. (2011). Sampling-based algorithms for optimal motion planning. Int. J. Rob. Res..

[28] Pérez-Higueras N., Caballero F., Merino L. (2018). Teaching Robot Navigation Behaviors to Optimal RRT Planners. Int. J. Soc. Robot..

[29] Koubaa A. (2018). Robot Operating System (ROS): The Complete Reference (Volume 3). Studies in Computational Intelligence.

[30] Pomerleau F., Colas F., Siegwart R., Magnenat S. (2013). Comparing ICP variants on real-world data sets. Auton. Robot..

[31] Pomerleau F., Magnenat S., Colas F., Liu M., Siegwart R. Tracking a Depth Camera: Parameter Exploration for Fast ICP. Proceedings of the 2011 IEEE/RSJ International Conference on Intelligent Robots and Systems.

